# Bridging Structure, Magnetism, and Disorder in Iron-Intercalated Niobium Diselenide, Fe_*x*_NbSe_2_, below *x* = 0.25

**DOI:** 10.1021/acs.jpcc.3c00870

**Published:** 2023-05-10

**Authors:** Matthew P. Erodici, Thuc T. Mai, Lilia S. Xie, Simon Li, Shannon S. Fender, Samra Husremović, Oscar Gonzalez, Angela R. Hight Walker, D. Kwabena Bediako

**Affiliations:** † Department of Chemistry, University of California, Berkeley, California 94720, United States; ‡ National Institute of Standards and Technology, Gaithersburg, Maryland 20899, United States; § Chemical Sciences Division, Lawrence Berkeley National Laboratory, Berkeley, California 94720, United States

## Abstract

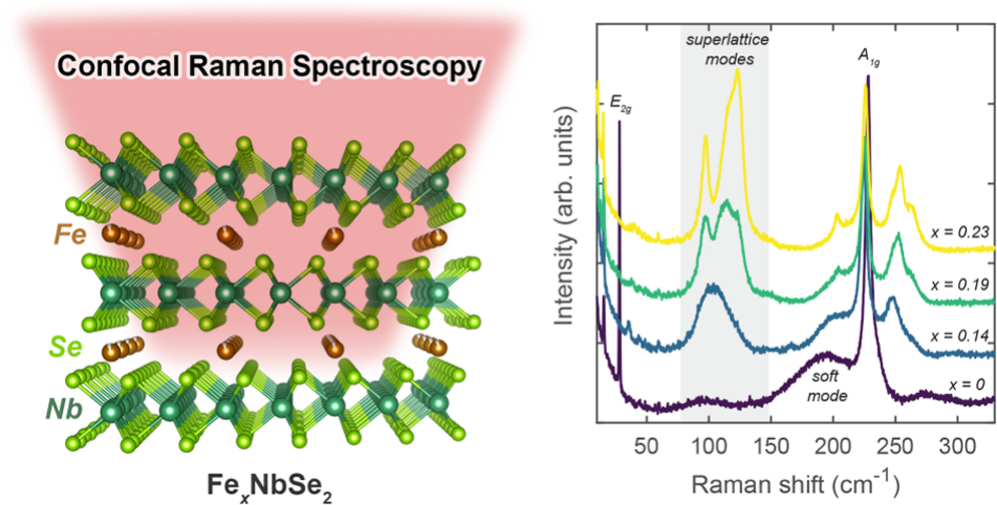

Transition-metal dichalcogenides (TMDs) intercalated with magnetic ions serve as a promising materials platform for developing next-generation, spin-based electronic technologies. In these materials, one can access a rich magnetic phase space depending on the choice of intercalant, host lattice, and relative stoichiometry. The distribution of these intercalant ions across given crystals, however, is less well defined—particularly away from ideal packing stoichiometries—and a convenient probe to assess potential longer-range ordering of intercalants is lacking. Here, we demonstrate that confocal Raman spectroscopy is a powerful tool for mapping the onset of intercalant superlattice formation in Fe-intercalated NbSe_2_ (Fe_*x*_NbSe_2_) for 0.14 ≤ *x* < 0.25. We use single-crystal X-ray diffraction to confirm the presence of longer-range intercalant superstructure and employ polarization-, temperature-, and magnetic field-dependent Raman measurements to examine both the symmetry of emergent phonon modes in the intercalated material and potential magnetoelastic coupling. Magnetometry measurements further indicate a correlation between the onset of magnetic ordering and the relative degree of intercalant superlattice formation. These results show Raman spectroscopy to be an expedient, local probe for mapping intercalant ordering in this class of magnetic materials.

## Introduction

Magnetically intercalated transition-metal dichalcogenides (TMDs) represent a promising class of emergent quantum materials for ultralow-power applications based on the manipulation of electron spin.^1−3^ In particular, iron-intercalated niobium-based dichalcogenides (Fe_*x*_NbS_2_ and Fe_*x*_NbSe_2_) are exciting material platforms for antiferromagnetic spintronics—with Fe_1/3_NbS_2_ hosting novel electrical switching responses, coupled to competing antiferromagnetic orders, that occur at unprecedented low current densities compared to other metallic antiferromagnetic systems.^1,4−9^ A recent study showed that the electrical switching response in Fe_*x*_NbS_2_ can be observed up to tens of microns away from the electrical stimulus, yet the mechanism behind the coherent spin transport required for this nonlocal effect is not well understood.^9^ Such length scales are much longer than typical electron scattering and spin decay lengths, raising questions about magnetoelastic coupling in these systems and the identity of the collective excitations that propagate the spin information. Understanding the origin of this behavior, therefore, requires a deeper understanding of the lattice dynamics in these systems. In addition, designing systems with analogous switching behavior closer to room temperature is desirable for any technological application. In this regard, the family of selenide analogues, Fe_*x*_NbSe_2_, are especially promising candidates since these compounds may exhibit higher magnetic ordering temperatures compared to the sulfides due to the higher spin–orbit coupling in heavier selenide compounds.^10−12^ However, these selenides remain underexplored relative to the sulfides and their transport and switching behavior have yet to be investigated.

A key ingredient that directs the nature of magnetic ordering in these materials more generally is the distribution of transition-metal intercalants within the host lattice.^2^ Raman scattering has been shown to be sensitive to the structure of intercalant superlattices within this family of magnetically intercalated TMDs.^13^ In particular, for several T*_x_*MS_2_ compounds (T = Fe, Cr; M = Nb, Ta), prominent Raman-active phonon modes emerge for *x* = 0.25 and 0.33, which are completely absent in the unintercalated host lattice.^3,13^ The onset of these modes coincides with the observation, via electron diffraction, of well-ordered 2*a*_0_ × 2*a*_0_ and √3*a*_0_ × √3*a*_0_ superlattices for *x* = 0.25 and 0.33, respectively (where *a*_0_ is the in-plane lattice constant for the host lattice).^3^ The correlation between these Raman modes and the underlying intercalant superlattice establishes Raman spectroscopy as a powerful and convenient tool to probe superlattice structure on the mesoscopic scale. While the Raman activity has been investigated for a small subset of these intercalated TMDs, particularly the sulfides, substantially less is known about the selenide variants and, in particular, for off-stoichiometric concentrations away from *x* = 0.25. Notably, the aforementioned switching behavior in Fe_*x*_NbS_2_ has been shown to be most pronounced when the compound is slightly off-stoichiometric from *x* = 1/3, hinting that disorder in the intercalant superlattices of these compounds may play a role in the coupling between electrical and magnetic orders.^4,8,9^ Here, we investigate how the Raman response evolves as a function of iron concentration in Fe_*x*_NbSe_2_, for 0.14 ≤ *x* < 0.25, finding a correlation between superlattice formation and iron occupancy. We also probe the magnetic behavior of these compounds and observe a maximum Néel temperature (*T*_N_) around 130 K as *x* approaches 0.25. Furthermore, we conduct polarization-, temperature-, and magnetic field-dependent Raman measurements to discern the symmetry of the observed phonon modes and investigate possible magnetoelastic couplings in this system.

## Methods

### Synthesis

Single crystals of Fe_*x*_NbSe_2_ were grown via chemical vapor transport with iodine as the transport agent. Elemental powders of Fe (99.9% Alfa Aesar), Nb (99.99%, Fisher Scientific), and Se (99.999%, Beantown Chemicals) were loaded in a 0.5:1:2 stoichiometric ratio, along with 1–2 mg cm^–3^ iodine, and sealed in evacuated quartz ampoules. The ampoules were loaded in a horizontal two-zone furnace with the hot end temperature set to 1100 °C and the cold end set to 1000 °C, ramping at ∼1 °C min^–1^. The gradient was held for 9 days, after which the hot end was cooled to 900 °C and subsequently kept 100 °C lower than the cold end to minimize condensation of iodine vapor on crystals in the cold end. The cold end temperature was then lowered to 700 °C, the gradient was held for 2 days, and then the power to the furnace was turned off. After cooling to room temperature, large, thin platelets (several millimeters in each lateral dimension) were found dispersed across the ampoule. The crystals were washed several times in either acetonitrile or ethanol to remove any excess iodine on the surface.

### Room-Temperature Raman Characterization

Confocal Raman spectra were collected on a Horiba LabRAM HR Evolution spectrometer with a 633 nm laser excitation source and the corresponding ultralow-frequency (ULF) notch filters, with an edge cutoff ∼10 cm^–1^. Samples were measured in backscattering configuration using a ×100 objective (N.A. 0.8) and measured with a laser power < 2 mW to avoid local heating effects. Measurements were performed using 10 s acquisition time and 10 accumulations, and all spectra were acquired with an 1800 groove mm^–1^ grating.

### Polarization-, Temperature-, and Magnetic Field-Dependent Raman Measurements

A triple-grating Raman spectrometer (Horiba JY T64000, 1800 mm^–1^ grating) coupled to a liquid-nitrogen-cooled CCD detector was used to collect the Raman data. The excitation wavelength was 632.8601 nm from a helium neon laser, and the spectra were collected in the 180° backscattering configuration. Raman spectra as a function of temperature and magnetic field were collected using an attoDRY cryostat (Attocube, Inc.), where the sample was zero-field-cooled and studied with a magnetic field-compatible objective (×50, N.A. 0.82). Ultra-broadband polarizers and achromatic half-wave plates were used to select and control polarization, including correcting for Faraday rotation in the objective under applied magnetic field. The laser power was ∼1.5 mW to avoid local heating, and integration times were ∼20 min.

### Scanning Electron Microscopy (SEM)–Energy-Dispersive X-ray Spectroscopy (EDX) Measurements

Scanning electron microscopy (SEM) and energy-dispersive X-ray spectroscopy (EDX) were conducted on an FEI Quanta SEM with an Oxford EDX detector using 20 keV accelerating voltage and 4 nA beam current.

### Single-Crystal X-ray Diffraction (SCXRD) Measurements

Suitable single crystals of Fe_*x*_NbSe_2_ (>1 mm × 1 mm) with relatively flat surfaces were selected for single-crystal X-ray diffraction (SCXRD). The *x* = 0.23 crystal was measured on a Rigaku XtaLab P200 diffractometer with Mo Kα radiation at 293 K. Data reduction and scaling and empirical absorption correction were performed in CrysAlis Pro. Using Olex2, the structure was solved with the SHELXT structure solution program using Intrinsic Phasing and refined against *F*^2^ on all data by full-matrix least squares using the SHELXL refinement package.^14−16^ The *x* = 0.14 and *x* = 0.19 crystals were measured on a Bruker AXS D8 Venture diffractometer under the same conditions, and the structures were solved within the accompanying Bruker APEX4 software program.

### Selected Area Electron Diffraction (SAED) Measurements

A single crystal of Fe_0.14_NbSe_2_ was mechanically exfoliated with scotch tape and transferred onto SiO_2_/Si substrates. Exfoliated flakes with thicknesses < 200 nm were identified using optical microscopy and atomic force microscopy. Target flakes were picked up with a poly(bisphenol-*A*-carbonate) (PC)/poly(dimethylsiloxane) (PDMS) stamp and transferred onto a 200-nm-thick amorphous Si_3_N_4_ membrane with 2-μm holes for transmission electron microscopy (TEM) imaging. Selected area electron diffraction (SAED) was performed on an FEI TitanX TEM operated at 80 kV, and data were collected along the [0001] zone axis of the Fe_0.14_NbSe_2_ flakes. The obtained patterns correspond to a 720 nm sample region, defined using a 40 μm diameter SAED aperture.

### Magnetometry Measurements

DC magnetization measurements as a function of temperature and applied field were carried out in a Quantum Design Physical Property Measurement System (PPMS) Dynacool with a 12 T magnet using the Vibrating Sample Magnetometer (VSM) option, with a detection limit of 10^–6^ emu. Crystals were mounted in polypropylene straws on crosses of cotton thread with the field parallel to the *c* axis.

### Thermoremanent Magnetization (TRM) Measurements

Thermoremanent magnetization (TRM) measurements were conducted in the Quantum Design PPMS with VSM option using the following protocol: (i) warming the sample to 400 K in zero magnetic field, (ii) setting the field to 1 T (applied along the *c* crystallographic axis), (iii) fast cooling to 20 K above the target temperature (at 10 K min^–1^), (iv) slow cooling to the target temperature (at 1 K min^–1^), (v) holding the sample in 1 T field for 1 h, and (vi) setting the field to 0 T and measuring the remanent magnetization in the sample over set time.

## Results and Discussion

### Crystal Structures of Fe_*x*_NbSe_2_ (*x* = 0.14, 0.19, and 0.23)

**Figure 1:**
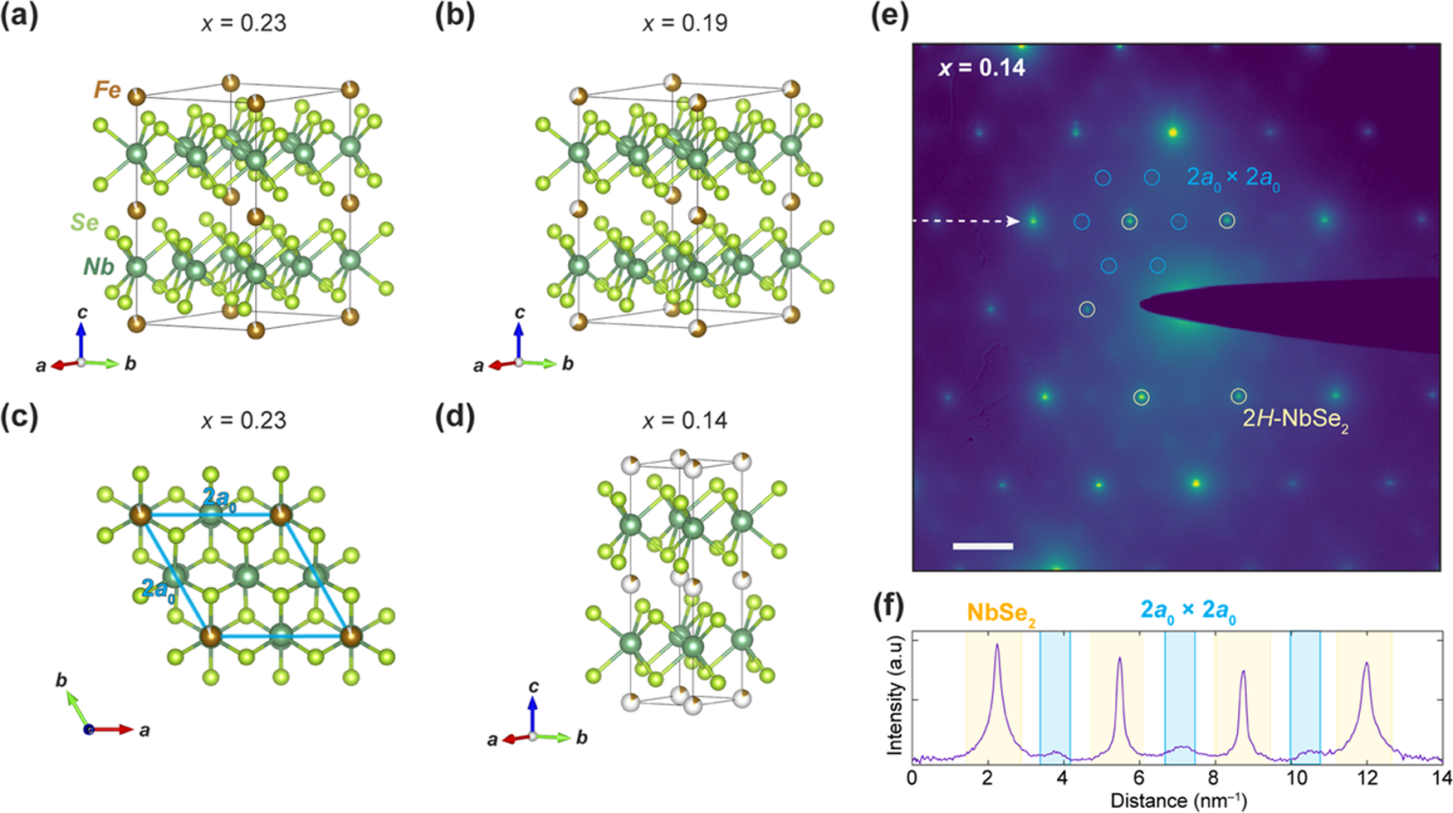
Fe_*x*_NbSe_2_ single-crystal structures viewed in standard orientation for (a) *x* = 0.23 and (b) *x* = 0.19, both of which exhibit a 2*a*_0_ × 2*a*_0_ Fe intercalant superlattice. (c) Top-down projection of *x* = 0.23 crystal structure, highlighting the 2*a*_0_ × 2*a*_0_ intercalant superlattice within the *ab* crystallographic plane. (d) Single-crystal structure for *x* = 0.14, in which the refined unit cell does not contain a periodic superlattice but rather Fe intercalants diffusely distributed among all octahedral interstitial sites. (e) Selected area electron diffraction (SAED) pattern of a Fe_0.14_NbSe_2_ flake. Diffraction peaks corresponding to 2*H*-NbSe_2_ are marked in yellow, while the faint 2*a*_0_ × 2*a*_0_ Fe superlattice reflections are circled in cyan. The SAED colormap is in logarithmic scale. Scale bar: 2 nm^–1^. (f) Horizontal line profile along the direction marked by the white arrow in (e). Higher-intensity peaks, shaded in yellow, correspond to 2*H*-NbSe_2_. The diffuse peaks, highlighted in cyan, mark the weak Fe superlattice reflections.

Single crystals of Fe_*x*_NbSe_2_ were synthesized via chemical vapor transport, and respective elemental compositions were determined via energy-dispersive X-ray spectroscopy (EDX). The range of Fe concentrations focused on in this work spanned from 0.14 ≤ *x* ≤ 0.23, highlighting samples with varying amounts of Fe deficiency relative to the stoichiometric amount of *x* = 0.25—which is associated with forming an ideal 2*a*_0_ × 2*a*_0_ superlattice. Single-crystal X-ray diffraction (SCXRD) data were obtained for single crystals at *x* = 0.14, 0.19, and 0.23 (Figure 1a–d), each yielding a hexagonal structure with the centrosymmetric space group **P**63/**mmc**. The out-of-plane lattice constant *c* was found to be 12.6011(9), 12.6174(8), and 12.6498(8) Å for *x* = 0.14, 0.19, and 0.23, respectively, which are all slightly larger than that for 2*H*-NbSe_2_ (*c*_0_ = 12.547(3) Å), suggesting a very minor expansion upon Fe intercalation.^17^ The in-plane lattice constant *a*, *b* for the *x* = 0.23 crystal was found to be 6.9151(4) Å, which is close to twice that of native 2*H*-NbSe_2_ (*a*_0_ = 3.4425(5) Å), and, similarly, the *a*, *b* lattice constant for the *x* = 0.19 sample was determined to be 6.9046(3) Å. For each of these two stoichiometries, the Fe intercalants occupy octahedral interstitial sites between the NbSe_2_ layers (Wyckoff position 2*a*), forming an ordered 2*a*_0_ × 2*a*_0_ superlattice (Figure 1c). In contrast, for the *x* = 0.14 structure, the in-plane lattice constant was found to be 3.4490(0) Å, and no superlattice was observed from SCXRD, as the Fe intercalants were best fit, on-average, to be weakly distributed among all octahedral sites. This global picture, however, does not preclude the possibility of Fe intercalants locally ordering on shorter length scales. Therefore, to confirm whether the *x* = 0.14 sample exhibits more local superlattice formation, selected area electron diffraction (SAED) data were taken on exfoliated flakes from the same single crystal (Figure 1e,f). Despite the presence of global intercalant disorder in Fe_0.14_NbSe_2_, diffuse 2*a*_0_ × 2*a*_0_ Fe superlattice reflections are observed in SAED, confirming the presence of local Fe ordering. The relative intensities of the superlattice reflections are substantially weaker and broadened by comparison to those of the NbSe_2_ host lattice, which still points to a relatively high degree of intercalant disorder, in line with the SCXRD structure.^18^

### Magnetic Properties of Fe_*x*_NbSe_2_ (*x* = 0.14, 0.19, and 0.23)

**Figure 2:**
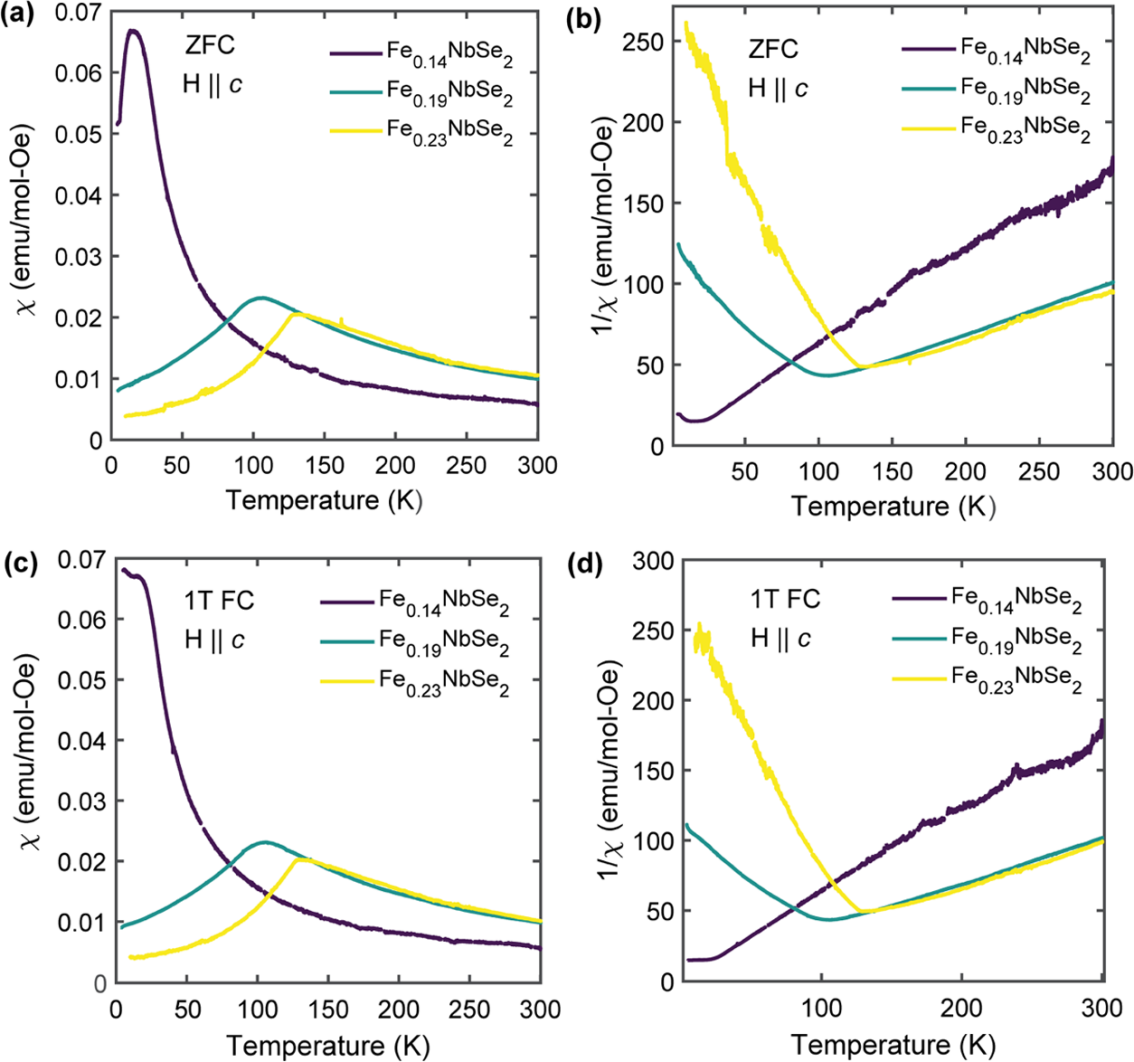
Magnetic susceptibility (χ) and inverse magnetic susceptibility (1/χ) data, obtained upon warming, for Fe_*x*_NbSe_2_ crystals of varying iron occupancy measured after (a, b) zero-field cooling (ZFC) and (c, d) 1T-field cooling (FC). The magnetic field was applied out-of-plane along the *c* crystallographic direction.

To evaluate the effect of Fe content on the magnetic ordering within this intercalation regime, magnetic susceptibility as a function of temperature was measured upon warming for the same representative Fe_*x*_NbSe_2_ crystals (Figure 2). As shown in Figure 2a,c, antiferromagnetic (AFM) transitions at 105 and 130 K can be clearly observed for Fe_0.19_NbSe_2_ and Fe_0.23_NbSe_2_, respectively, with negligible bifurcation between field-cooled (FC) and zero-field-cooled (ZFC) traces. The Fe_0.14_NbSe_2_ sample, on the other hand, exhibits paramagnetic behavior down to approximately 20 K followed by a broad transition below 20 K. There is also a substantial bifurcation in FC vs ZFC behavior for Fe_0.14_NbSe_2_ (Figure 2a vs Figure 2c), which suggests possible glassy behavior or the presence of a more complex spin-dilute phase in the material.^10−12^ Thermoremanent magnetization (TRM) measurements—which are sensitive to glassy dynamics^19,20^—were subsequently conducted on the Fe_0.14_NbSe_2_ sample to probe for such glassy behavior (Figure S3). The onset of a nonzero remanent magnetization for temperatures ≤ 10 K, with a concomitant slow decay in magnetization signal over time, corroborates the presence of a spin-glass-like phase below 20 K.^19,20^ Overall, the lack of robust AFM ordering for Fe_0.14_NbSe_2_ likely stems from a higher degree of structural disorder, consistent with the SCXRD measurements, which would disrupt the uniformity of the magnetic exchange interactions among the spin-bearing Fe intercalants.

Regarding other magnetic properties of these compounds, the in-plane magnetic susceptibility for the Fe_0.23_NbSe_2_ crystal (Figure S2) was found to be significantly weaker than the respective out-of-plane magnetic susceptibility, signaling strong magnetocrystalline anisotropy (MCA) for the out-of-plane *c* direction in this material. This MCA effect arises due to unquenched orbital angular momentum, stemming from the unevenly occupied e_g_ set in the d-electron configuration of octahedral high-spin Fe^2+^, and is consistent with other Fe-intercalated TMDs.^2,3,10^ The high-spin Fe^2+^ configuration is a valid approximation of the spin state of the Fe intercalants in this material because the expected effective moment for octahedral high-spin Fe^2+^ (μ_eff_ = 4.90μ_B_) matches closely with the experimental μ_eff_ = 5.11μ_B_ calculated for the Fe_0.23_NbSe_2_ sample, which was obtained based on the slope of a linear fit to the paramagnetic regime within the respective inverse susceptibility (1/χ) vs temperature plot (Figure 2b).^21^ The Curie–Weiss temperatures for Fe_0.14_NbSe_2_, Fe_0.19_NbSe_2_, and Fe_0.23_NbSe_2_, moreover, were extracted from the x-intercepts of the same corresponding linear fits and estimated to be −2, −11, and −15 K, respectively—in line with short-range antiferromagnetic-type interactions.^21^ Taken together, increasing the Fe concentration for this range of stoichiometries manifests in higher magnetic ordering temperatures—with Fe_0.23_NbSe_2_ exhibiting the highest Néel temperature (*T*_N_) at 130 K. This trend in increasing ordering temperature correlates with the increased degree of intercalant superlattice formation, based on SCXRD, as Fe content increases, which is sensible given that a well-ordered intercalant superstructure should give rise to more uniform and robust magnetic exchange interactions between magnetic ions.

### Confocal Raman Spectroscopy

**Figure 3:**
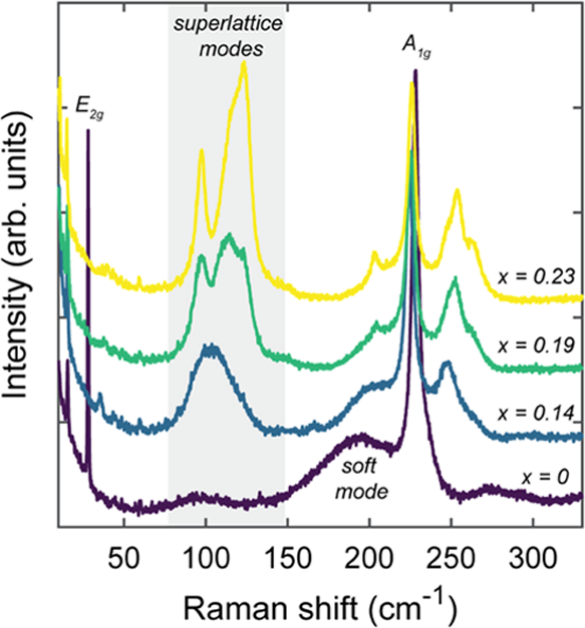
Raman spectra of representative Fe_*x*_NbSe_2_ crystals taken under ambient conditions and acquired with 633 nm laser excitation. The predominant Raman-active modes for the parent compound, 2*H*-NbSe_2_, are labeled in black, and the frequency region consistent with phonon modes for an intercalant superlattice is shaded in gray.

Raman scattering studies were performed to further characterize and differentiate the structural properties between Fe_*x*_NbSe_2_ samples (Figure 3). For the host lattice compound 2*H*-NbSe_2_ (*x* = 0), which has *D*_6*h*_^4^ point group symmetry, the optical phonons at the Brillouin zone (BZ) center have the following irreducible representation: Γ = A_1g_ + 2A_2u_ + 2B_2g_ + B_1u_ + E_1g_ + 2E_2g_ + 2E_1u_ + E_2u_.^22−25^ Of these, there are four Raman-active modes, namely, A_1g_ + E_1g_ + 2E_2g_. In Raman backscattering configuration along the *ZZ* direction, in which these measurements were conducted, the E_1g_ mode should not be detected because the E_1g_ Raman tensor (R_E_1g__) for the *P*6_3_/*mmc* space group does not contain nonzero polarizability matrix elements for this sample geometry—thereby leaving only A_1g_ and E_2g_ modes to be observed.^22,24−26^ Therefore, for *x* = 0, the sharp mode at 28 cm^–1^ was assigned to the well-known interlayer E_2g_ shear mode for 2*H*-NbSe_2_ and the peak at 228 cm^–1^ was assigned to the corresponding intralayer A_1g_ mode.^26−28^ Another intralayer E_2g_ mode is expected for 2*H*-NbSe_2_ at 237 cm^–1^ though this feature was not readily apparent with 633 nm excitation; however, upon switching to 532 nm excitation, this peak was later detected (Figure S4)—consistent with prior literature comparing Raman scattering of 2*H*-NbSe_2_ across different excitation wavelengths.^27^ The broad feature centered around 192 cm^–1^ for *x* = 0 was attributed to a soft phonon mode, related to charge–density wave interactions, which arises from a second-order Raman scattering process of two Brillouin zone-edge longitudinal acoustic phonons.^27,29−31^

In the case of the Fe-intercalated materials, several changes to the Raman spectrum can be seen, compared to that of the 2*H*-NbSe_2_ host lattice. The interlayer shear mode for NbSe_2_ at 28 cm^–1^ disappears with Fe intercalation, which can be attributed to Fe intercalants forming covalent bonds with the NbSe_2_ host lattice, thus precluding the NbSe_2_ layers—originally held together by weak van der Waals (vdW) forces—from exhibiting any strong interlayer shearing motions. Most significantly, a series of new phonon modes emerge in the intercalated compounds in the spectral region below 150 cm^–1^ as well as adjacent to the host lattice NbSe_2_ A_1g_ Raman peak. As the Fe concentration increases, the new modes between 70 and 150 cm^–1^ (gray-shaded region in Figure 3) evolve from one broad hump for *x* = 0.14 to three resolvable peaks centered at 97, 113, and 125 cm^–1^ for *x* = 0.23.^25^ The frequency range of these features appears consistent with phonon modes attributed to intercalant superlattice formation in other T_*x*_MCh_2_ compounds.^2,13,25^ Additionally, the sharp linewidths for the superlattice features in the *x* = 0.23 sample are consistent with a clear 2*a*_0_ × 2*a*_0_ intercalant superstructure in the Fe_0.23_NbSe_2_ crystal structure. The intermediate but incomplete growth of superlattice Raman peaks for *x* = 0.19 crystal, on the other hand, suggests the formation of a defective superlattice with missing intercalants, compared to *x* = 0.23, which would also be in conjunction with a clear but weaker AFM transition for *x* = 0.19 in the magnetic susceptibility data (Figure 2a). The nature of the superstructure in the *x* = 0.19 compound may, therefore, be more akin to a patchwork of locally ordered/disordered intercalant domains throughout the lattice, rather than a perfect 2*a*_0_ × 2*a*_0_ network, which would reflect the Fe occupancy being significantly less than the ideal packing ratio of *x* = 0.25.^3^

Aside from the new superlattice features below 150 cm^–1^, the higher-frequency emergent phonon modes centered at 248, 253, and 263 cm^–1^ likely arise, in part, from newly formed Fe–Se vibrations. These peak positions are consistent with the Raman frequency range of Fe–Se stretching vibration modes observed in other crystalline systems.^17,32^ These features could also stem from Brillouin zone-folding effects, which arise when a structure of higher crystal symmetry is superimposed with that of a lower crystal symmetry, potentially allowing phonons at high-symmetry points of one BZ to map onto different high-symmetry points of the other.^25^ Therefore, for a Fe_0.23_NbSe_2_ structure with a 2*a*_0_ × 2*a*_0_ intercalant supercell, zone-edge optical phonons located at the *M* point of the host lattice Brillouin zone (*M*_NbSe_2__) can now be folded onto the zone-center Γ point of the superlattice BZ (Γ_SL_).^25^ This new crystal symmetry, therefore, folds these optical phonons with originally high crystal momenta onto a new symmetry point with lower crystal momentum, thereby allowing incident photons with very little inherent momentum to interact with and scatter off these excited vibrational modes.^24,25^ Notably, the modes in this energy region appear more well resolved as Fe concentration increases—similar to the evolution of the superlattice-related features below 150 cm^–1^—which would correlate with a progressively higher degree of intercalant ordering and thus a higher degree of potential zone-folding effects.

In regard to other changes, there is an additional redshifting of the NbSe_2_-related A_1g_ mode upon Fe intercalation, with the A_1g_ peak shifting from 228.0 cm^–1^ for *x* = 0–224.5 cm^–1^ for *x* = 0.14. This frequency red shift could be due to weakened polarizability around the Nb–Se vibrational mode as electron density gets redistributed away from Nb–Se bonds toward some Fe–Se covalency upon initial Fe intercalation. Interestingly, this A_1g_ peak blue-shifts back toward higher frequencies as more Fe is introduced, shifting from 224.5 cm^–1^ for *x* = 0.14–226.0 cm^–1^ at *x* = 0.23. This diverging effect could stem from an increasingly higher filling level of the host lattice conduction band, which is primarily of Nb *d*_*z*2_ character,^2^ thereby restoring some electron density around Nb–Se vibrational modes. A similar frequency blue shift is observed for the Fe–Se-related modes above 240 cm^–1^ as *x* increases from *x* = 0.14 to *x* = 0.23, which may also be rationalized by a stiffening of the Fe–Se-related spring constant due to increased charge transfer from intercalated Fe^2+^ ions to the Nb–Se host layer.^33^

### Polarization-Dependent Raman Spectroscopy

**Figure 4:**
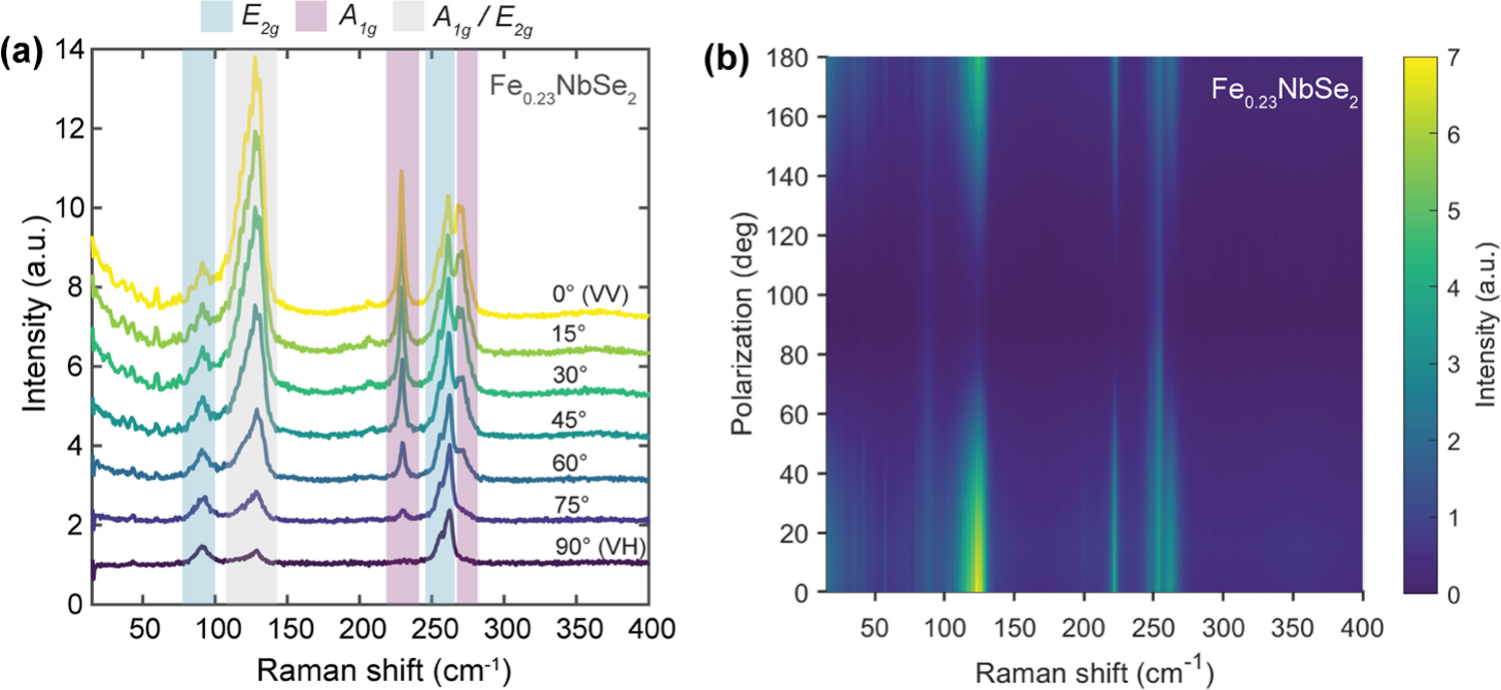
(a) Polarized Raman data for Fe_0.23_NbSe_2_ single crystal measured by detecting the component of Raman scattered light that is rotated by θ° relative to the linear polarization axis of the incident light. Here, 0° denotes parallel (VV) configuration and 90° denotes cross (VH) configuration. (b) Surface intensity plot of polarized Raman data acquired for the 0–180° polarization range, highlighting the disappearance of modes with *A*_1*g*_ symmetry when measuring in cross-configuration (90°). All data were collected at 1.6 K and acquired with a 633 nm excitation wavelength.

To better understand the symmetry of emergent Raman modes in the intercalated Fe_0.23_NbSe_2_ system, polarized Raman measurements were carried out by measuring Raman intensity as a function of rotation angle between incident and scattered light polarization vectors (Figure 4). Two linear polarizers were set parallel to each other—one in the incoming laser excitation path and the other in the spectrometer detection path—and a half-wave plate was mounted directly in front of the latter polarizer. Rotating the half-wave plate by θ/2 allowed for the detection of Raman scattered light that was effectively rotated by θ with respect to the polarization vector of the incident laser light.^34^ For the space group *P*6_3_/*mmc* and specific Wyckoff positions occupied in the Fe_0.23_NbSe_2_ crystal structure, the symmetry-adapted phonon modes (SAMs) expected at the zone-center Γ_SL_ point are: Γ_acoustic_ = A_2u_ + E_1u_ and Γ_optical_ = 4A_1g_ + A_1u_ + 2A_2g_ + 5A_2u_ + 5B_1g_ + 2B_1u_ + B_2g_ + 5B_2u_ + 5E_1g_ + 7E_1u_ + 7E_2g_ + 6E_2u_.^35−38^ Based on the optical phonons present, the expected Raman-active modes are 4A_1g_ + 5E_1g_ + 7E_2g_.^35−38^ As in the unpolarized Raman experiment, the backscattering measurement geometry along the *ZZ* direction precludes the detection of the E_1g_ modes for materials with this space group symmetry.^38^

For the A_1g_ and E_2g_ Raman modes, the relative orientation between the incident and scattered light polarization vectors determines which modes should appear most intensely in Raman scattering at a given angle. In parallel configuration, in which the polarization directions of the incident and scattered light are the same (i.e., *XX* or *YY*) and the relative angle between them is 0°, only modes that contain a nonzero polarizability matrix element for α_*xx*_ and α_*yy*_ in the corresponding Raman tensors will be detected. Therefore, when in this sampling configuration, both types of modes should be observed, as the respective Raman tensors (*R*_A_1g__ and *R*_E_2g__) under *P*6_3_/*mmc* symmetry satisfy this criterion.^38^ Similarly, in cross-configuration, in which the relative polarization vectors are orthogonal (90°) to one another (i.e., *XY* or *YX*), only phonon modes that contain a nonzero polarizability matrix element for α_*xy*_ and α_*yx*_ should appear strongly in Raman scattering. Consequently, only E_2g_ modes should persist in this sampling geometry because only the E_2g_ tensor (*R*_E_2g__) obeys this condition under *P*6_3_/*mmc* symmetry.^38^

Operating under this framework, we can discern the A_1g_ or E_2g_ character of the emergent Raman modes in the intercalated systems. For Fe_0.23_NbSe_2_, all A_1g_ and E_2g_ modes appear together in parallel configuration (0°), consistent with the confocal Raman experiment. However, as the relative angle between the incident and scattered polarization vectors changes from 0 to 90°, we observe the attenuation of the features at 125, 226, and 263 cm^–1^ and the prevalence of the remaining modes at 97, 248, and 253 cm^–1^. Therefore, based on the governing polarization selection rules, the former set of phonon modes can be ascribed to A_1g_ symmetry while the latter can be assigned to E_2g_ symmetry. There is some remaining intensity in the spectral region for the superlattice modes around 125 cm^–1^ under cross-polarization, which could be due to a convolution of A_1g_ and E_2g_ modes pertaining to possibly nearly degenerate out-of-plane and in-plane intercalant superlattice vibrations.^13^

### Temperature- and Magnetic Field-Dependent Raman Spectroscopy

**Figure 5:**
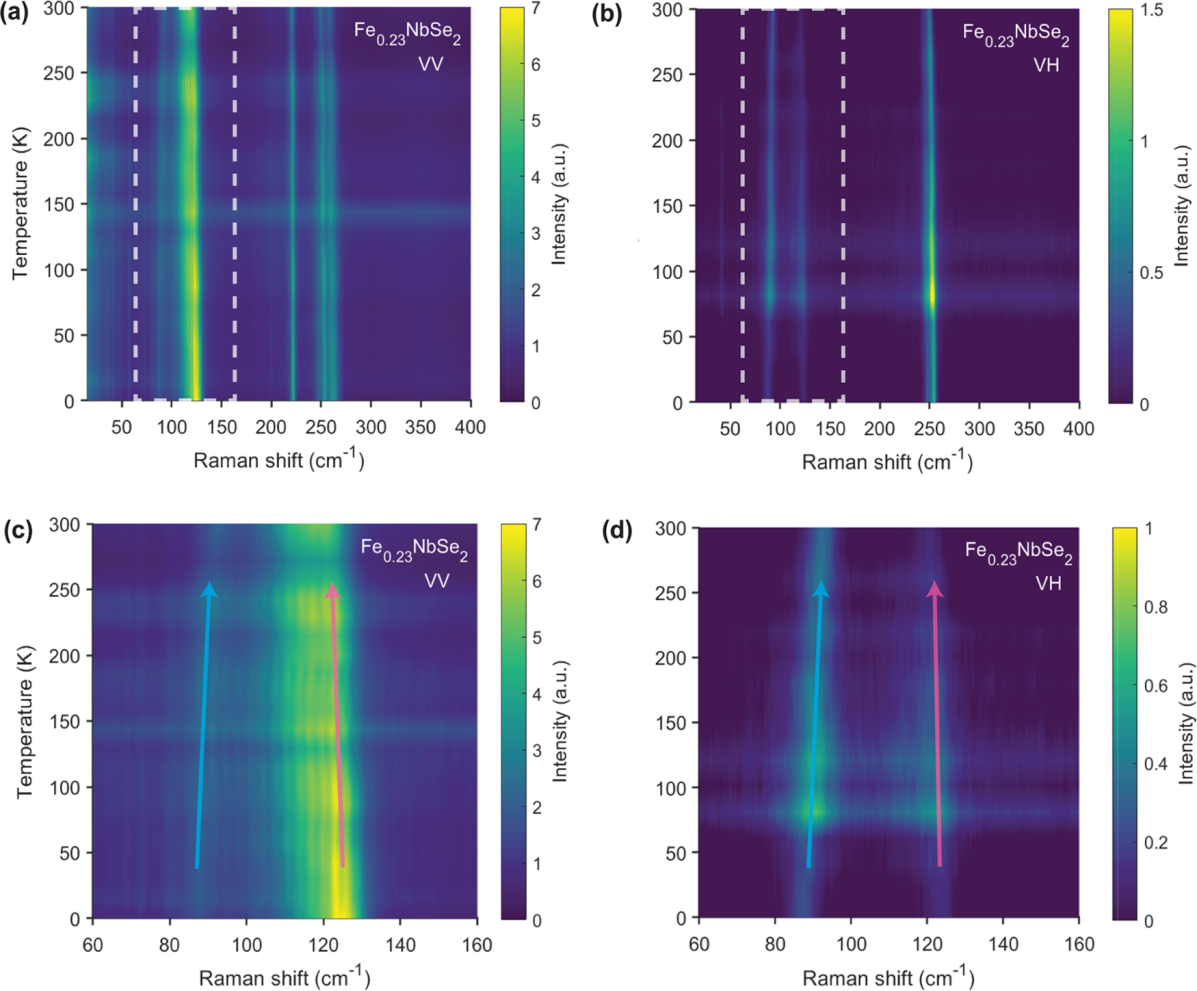
Variable-temperature Raman data for a Fe_0.23_NbSe_2_ single crystal when measured in (a, c) parallel (VV) and (b, d) cross (VH)-polarization configurations. (c, d) Magnified view of the spectral region in which intercalant superlattice phonon modes are expected to be present (dashed white box in (a) and (b)). The *E*_2*g*_ mode below 100 cm^–1^ shows an anomalous blue shift with increasing temperature (denoted by the blue arrow). In comparison, the *A*_1*g*_ mode above 110 cm^–1^ red-shifts with increasing temperature as expected due to increased phonon anharmonicity (denoted by red arrow).

**Figure 6:**
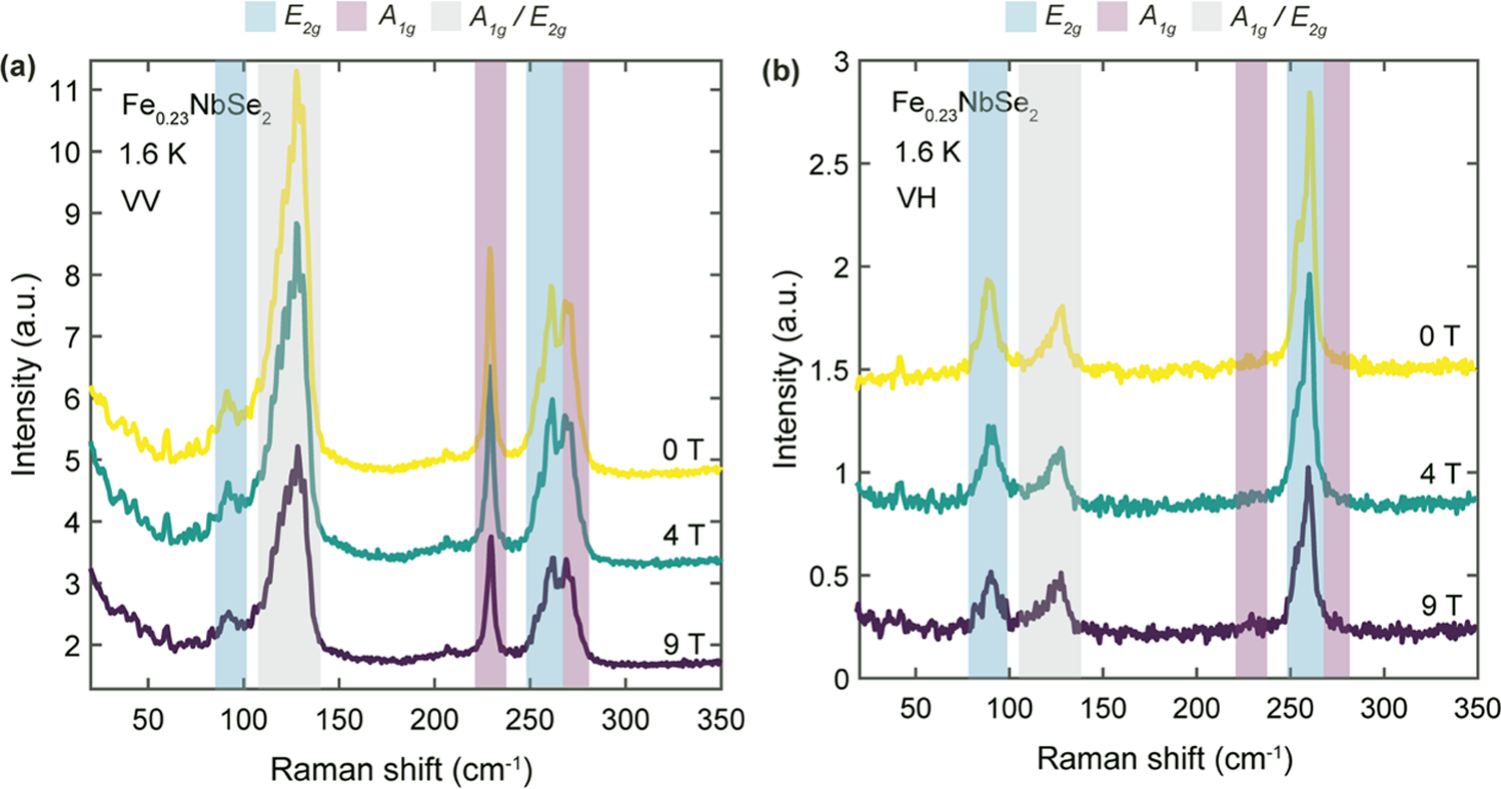
Magneto-Raman spectra for Fe_0.23_NbSe_2_ single crystal measured in (a) parallel (VV) and (b) cross (VH)-polarization configurations. The shaded regions denote the symmetry of the respective phonon modes, as determined by the polarized Raman measurements. The data were acquired below the magnetic ordering temperature, and the magnetic field was applied along the *c* crystallographic axis. All spectra were measured in Faraday geometry and with 633 nm excitation wavelength.

To probe whether strong magnetoelastic coupling was present in the system, Raman scattering as a function of temperature (Figure 5) and applied magnetic field (Figure 6) was measured on the same Fe_0.23_NbSe_2_ crystal. The temperature-dependent Raman spectra, for both parallel and cross configurations (Figure 5), reveal no major transitions before or after crossing the Néel temperature at 130 K, unlike other magnetic systems that do exhibit significant magnetoelastic coupling.^39−42^ Most of the phonon modes were found to red-shift as expected with increasing temperature, due to conventional phonon anharmonicity and thermal expansion effects at higher temperatures.^43^ However, one of the superlattice E_2g_ modes was found to unexpectedly blue-shift with increasing temperature (Figure 5d), with a peak near 90 cm^–1^ at 1.6 K that shifts to 96 cm^–1^ at 300 K, thereby appearing stiffer rather than softer. While the definitive origin for this phonon behavior remains unclear, one possible cause for this anomalous temperature dependence could be differences in volumetric expansion between in-plane and out-of-plane intercalant superlattice modes.^43−45^

Magnetic field-dependent Raman spectra were acquired at 1.6 K, with applied field along the magnetic easy axis, to probe the presence of antiferromagnetic magnons as well as strong magnetoelastic coupling at large in the crystal. We do not observe any signatures of antiferromagnetic magnons, which would be characterized by an applied magnetic field (*H*_0_) causing a Zeeman splitting among degenerate magnon normal modes, leading to two magnon peaks symmetrically offset above and below the original peak energy in the Raman spectrum.^42,46^ In addition, unlike systems with strong magnetoelastic coupling such as FePS_3_, which undergoes significant changes in the unit cell and Brillouin zone upon magnetic ordering and, concomitantly, exhibits emergent phonon modes due to zone-folding effects,^40,42^ there does not appear to be such a symmetry effect in Fe_0.23_NbSe_2_ when it orders antiferromagnetically. This lack of an effect below the Néel temperature may be due to Fe_0.23_NbSe_2_ not undergoing a change in symmetry to the superlattice Brillouin zone above and below the magnetic transition, as is the case with FePS_3_.^40^ As such, at least within this intercalation regime for *x* < 0.25, there is no observation of significant magnetoelastic effects as probed by Raman scattering. Future experimental work on more Fe-rich intercalation compounds in the *x* = 0.33 regime, which possess a different space group symmetry and intercalant superlattice structure compared to the *x* = 0.25 analogues, may reveal whether such systems are more amenable to hosting such phenomena—potentially due to more compatible zone-folding effects upon magnetic ordering from the interplay between crystal symmetry and relative antiferromagnetic spin configurations.

## Conclusions

Overall, the structural and magnetic properties of Fe_*x*_NbSe_2_ for off-stoichiometric iron amounts *x* = 0.14, 0.19, and 0.23 suggest the emergence of intercalant superlattice formation in iron-deficient samples as low as *x* = 0.19. These superlattices can be conveniently probed by confocal Raman scattering, and the symmetry of these new modes can be distinguished by angle-polarized Raman spectroscopy. The nature of these superlattices in Fe-deficient compounds between 0.19 ≤ *x* ≤ 0.23 is likely based on a progressively less defective *2a*_0_ × *2a*_0_ superstructure approaching the ideal packing at *x* = 0.25. In Raman scattering, three intercalant superlattice modes emerge at 97, 113, and 125 cm^–1^ at *x* = 0.19 and become more intense and well resolved as Fe concentration increases to *x* = 0.23. Future studies will aim to map out the Raman-active modes for higher intercalation amounts in the Fe_*x*_NbSe_2_ family, namely, for 0.25 ≤ *x* ≤ 0.33 and for *x* > 0.33, in which a crossover to a noncentrosymmetric *P*6_3_22 space group is expected and thus a √3*a*_0_ × √3*a*_0_ superlattice should be observed.^2,13,24,33^ Assessing the extent of magnetoelastic coupling in the intercalation regime near *x* = 0.33 would also lend valuable insight as to whether fundamentally new phonons can emerge from zone-folding and couple directly with the spin ordering in the system. Furthermore, examining how the modes for a different superlattice prevail within Fe_*x*_NbSe_2_ will play a key role in screening for different superlattice formation within a given crystal, providing a road map for probing and exploiting local disorder and coexisting structural domains within this magnetically intercalated TMD family.

## Supporting Information Available

Additional magnetometry measurements, Raman spectra, and single-crystal XRD data (PDF)

## References

[ref1] Nair N. L.; Maniv E.; John C.; Doyle S.; Orenstein J.; Analytis J. G. Electrical switching in a magnetically intercalated transition metal dichalcogenide. Nat. Mater. 2020, 19, 153–157. 10.1038/s41563-019-0518-x. 31685945

[ref2] Xie L. S.; Husremović S.; Gonzalez O.; Craig I. M.; Bediako D. K. Structure and Magnetism of Iron-and Chromium-Intercalated Niobium and Tantalum Disulfides. J. Am. Chem. Soc. 2022, 144, 9525–9542. 10.1021/jacs.1c12975. 35584537

[ref3] Husremović S.; Groschner C. K.; Inzani K.; Craig I. M.; Bustillo K. C.; Ercius P.; Kazmierczak N. P.; Syndikus J.; Van Winkle M.; Aloni S.; et al. Hard Ferromagnetism Down to the Thinnest Limit of Iron-Intercalated Tantalum Disulfide. J. Am. Chem. Soc. 2022, 144, 12167–12176. 10.1021/jacs.2c02885. 35732002

[ref4] Maniv E.; Nair N. L.; Haley S. C.; Doyle S.; John C.; Cabrini S.; Maniv A.; Ramakrishna S. K.; Tang Y.-L.; Ercius P.; et al. Antiferromagnetic switching driven by the collective dynamics of a coexisting spin glass. Sci. Adv. 2021, 7, eabd8452 10.1126/sciadv.abd8452. 33523993 PMC7793592

[ref5] Maniv E.; Murphy R. A.; Haley S. C.; Doyle S.; John C.; Maniv A.; Ramakrishna S. K.; Tang Y.-L.; Ercius P.; Ramamoorthy R.; et al. Exchange bias due to coupling between coexisting antiferromagnetic and spin-glass orders. Nat. Phys. 2021, 17, 525–530. 10.1038/s41567-020-01123-w.

[ref6] Haley S. C.; Weber S. F.; Cookmeyer T.; Parker D. E.; Maniv E.; Maksimovic N.; John C.; Doyle S.; Maniv A.; Ramakrishna S. K.; et al. Half-magnetization plateau and the origin of threefold symmetry breaking in an electrically switchable triangular antiferromagnet. Phys. Rev. Res. 2020, 2, 043020 10.1103/PhysRevResearch.2.043020.

[ref7] Weber S. F.; Neaton J. B. Origins of anisotropic transport in the electrically switchable antiferromagnet Fe_1/3_NbS_2_. Phys. Rev. B 2021, 103, 214439 10.1103/PhysRevB.103.214439.

[ref8] Wu S.; Xu Z.; Haley S. C.; Weber S. F.; Acharya A.; Maniv E.; Qiu Y.; Aczel A. A.; Settineri N. S.; Neaton J. B.; et al. Highly Tunable Magnetic Phases in Transition-Metal Dichalcogenide Fe1/3+δNbS2. Phys. Rev. X 2022, 12, 021003 10.1103/PhysRevX.12.021003.

[ref9] Haley S. C.; Maniv E.; Wu S.; Cookmeyer T.; Torres-Londono S.; Aravinth M.; Maksimovic N.; Moore J.; Birgeneau R. J.; Analytis J. G. Long-range, Non-local Switching of Spin Textures in a Frustrated Antiferromagnet. 2021, arXiv:2111.09882. arXiv.org e-Print archive. https://doi.org/10.48550/arXiv.2111.09882. 10.1038/s41467-023-39883-7 PMC10403493 37542056

[ref10] Hillenius S. J.; Coleman R. V. Magnetic susceptibility of iron-doped 2H-NbSe2. Phys. Rev. B 1979, 20, 4569–4576. 10.1103/PhysRevB.20.4569.

[ref11] Garvin J. F. Jr; Morris R. C. Transport properties and magnetic ordering in iron-doped NbSe_2_. Phys. Rev. B 1980, 21, 2905 10.1103/PhysRevB.21.2905.

[ref12] Whitney D. A.; Fleming R. M.; Coleman R. V. Magnetotransport and superconductivity in dilute Fe alloys of NbSe_2_, TaSe_2_, and TaS_2_. Phys. Rev. B 1977, 15, 3405 10.1103/PhysRevB.15.3405.

[ref13] Fan S.; Neal S.; Won C.; Kim J.; Sapkota D.; Huang F.; Yang J.; Mandrus D. G.; Cheong S.-W.; Haraldsen J. T.; Musfeldt J. L. Excitations of intercalated metal monolayers in transition metal dichalcogenides. Nano Lett. 2021, 21, 99–106. 10.1021/acs.nanolett.0c03292. 33264028

[ref14] Dolomanov O. V.; Bourhis L. J.; Gildea R. J.; Howard J. A.; Puschmann H. OLEX2: a complete structure solution, refinement, and analysis program. J. Appl. Crystallogr. 2009, 42, 339–341. 10.1107/S0021889808042726.

[ref15] Sheldrick G. M. SHELXT-Integrated space-group and crystal-structure determination. Acta Crystallogr., Sect. A: Found. Adv. 2015, 71, 3–8. 10.1107/S2053273314026370. 25537383 PMC4283466

[ref16] Sheldrick G. M. Crystal structure refinement with SHELXL. Acta Crystallogr., Sect. C: Struct. Chem. 2015, C71, 3–8. 10.1107/S2053229614024218. PMC4294323 25567568

[ref17] Naik S.; Pradhan A.; Mishra A.; Samal S. L. Evolution of Structural Properties in Fe Intercalated 2H-NbSe_2_: Phase Transformation Induced by Strong Host–Guest Interaction. J. Phys. Chem. C 2022, 126, 13762–13773. 10.1021/acs.jpcc.2c03511.

[ref18] Scholz G. A.; Frindt R. F.; Curzon A. E. Electron Diffraction Investigation of the Ag_*x*_TaS_2_ System II. Superlattices, Structure, and Charge Density Waves in Ag_*x*_TaS_2_. Phys. State Solidi (A) 1982, 72, 375–390. 10.1002/pssa.2210720139.

[ref19] Mitchler P. D.; Roshko R. M.; Ruan W. Non-equilibrium relaxation dynamics in the spin glass and ferromagnetic phases of CrFe. Philos. Mag. B 1993, 68, 539–550. 10.1080/13642819308217933.

[ref20] Samarakoon A.; Sato T. J.; Chen T.; Chern G.-W.; Yang J.; Klich I.; Sinclair R.; Zhou H.; Lee S.-H. Aging, memory, and nonhierarchical energy landscape of spin jam. Proc. Natl. Acad. Sci. U.S.A. 2016, 113, 11806–11810. 10.1073/pnas.1608057113. 27698141 PMC5081640

[ref21] Mugiraneza S.; Hallas A. M. Tutorial: a beginner’s guide to interpreting magnetic susceptibility data with the Curie-Weiss law. Commun. Phys. 2022, 5, 95 10.1038/s42005-022-00853-y.

[ref22] McMullan W. G.; Irwin J. C. Raman scattering from 2H and 3R–NbS_2_. Solid State Commun. 1983, 45, 557–560. 10.1016/0038-1098(83)90426-X.

[ref23] Nakashima S.; Tokuda Y.; Mitsuishi A.; Aoki R.; Hamaue Y. Raman scattering from 2H-NbS_2_ and intercalated NbS_2_. Solid State Commun. 1982, 42, 601–604. 10.1016/0038-1098(82)90617-2.

[ref24] Nagao K.; Koyano M.; Katayama S.; Yamamura Y.; Tsuji T. Raman scattering from intercalation compounds FexNbS2 under high pressure. Phys. State Solidi (B) 2001, 223, 281–285. 10.1002/1521-3951(200101)223:1<281::AID-PSSB281>3.0.CO;2-Z.

[ref25] Pereira C. M.; Liang W. Y. Raman study of iron-intercalated niobium selenide. J. Phys. C: Solid State Phys. 1985, 18, 6075–6082. 10.1088/0022-3719/18/32/019.

[ref26] Wang C. S.; Chen J. M. Raman spectrum of metallic layered compound NbSe_2_. Solid State Commun. 1974, 14, 1145–1148. 10.1016/0038-1098(74)90292-0.

[ref27] Hill H. M.; Rigosi A. F.; Krylyuk S.; Tian J.; Nguyen N. V.; Davydov A. V.; Newell D. B.; Hight Walker A. R. Comprehensive optical characterization of atomically thin NbSe_2_. Phys. Rev. B 2018, 98, 165109 10.1103/PhysRevB.98.165109. PMC6459197 30984898

[ref28] He R.; van Baren J.; Yan J.-A.; Xi X.; Ye Z.; Ye G.; Lu I.-H.; Leong S. M.; Lui C. H. Interlayer breathing and shear modes in NbSe_2_ atomic layers. 2D Mater. 2016, 3, 031008 10.1088/2053-1583/3/3/031008.

[ref29] Xi X.; Zhao L.; Wang Z.; Berger H.; Forró L.; Shan J.; Mak K. F. Strongly enhanced charge-density-wave order in monolayer NbSe_2_. Nat. Nanotechnol. 2015, 10, 765–769. 10.1038/nnano.2015.143. 26192206

[ref30] Tsang J. C.; Smith J. E. Jr; Shafer M. W. Raman spectroscopy of soft modes at the charge-density-wave phase transition in 2H-NbSe2. Phys. Rev. Lett. 1976, 37, 1407–1410. 10.1103/PhysRevLett.37.1407.

[ref31] Tsang J. C.; Smith J. E. Jr; Shafer M. W. Effect of charge density wave fluctuations on the frequencies of optic phonons in 2H-TaSe_2_ and-NbSe_2_. Solid State Commun. 1978, 27, 145–149. 10.1016/0038-1098(78)90820-7.

[ref32] Xu J.; Li W.; Zhang B.; Zha L.; Hao W.; Hu S.; Yang J.; Li S.; Gao S.; Hou Y. Free-standing 2D non-van der Waals antiferromagnetic hexagonal FeSe semiconductor: halide-assisted chemical synthesis and Fe2+ related magnetic transitions. Chem. Sci. 2021, 13, 203–209. 10.1039/D1SC04122C. 35059168 PMC8694323

[ref33] Koyano M.; Watanabe H.; Yamamura Y.; Tsuji T.; Katayama S. Magnetic and Raman scattering studies on intercalation compounds Fe_x_NbS_2_. Mol. Cryst. Liq. Cryst. Sci. Technol., Sect. A 2000, 341, 33–38. 10.1080/10587250008026113.

[ref34] Lacinska E. M.; Furman M.; Binder J.; Lutsyk I.; Kowalczyk P. J.; Stepniewski R.; Wysmolek A. Raman Optical Activity of 1T-TaS_2_. Nano Lett. 2022, 22, 2835–2842. 10.1021/acs.nanolett.1c04990. 35369696 PMC9011401

[ref35] Aroyo M. I.; Perez-Mato J. M.; Orobengoa D.; Tasci E.; de la Flor G.; Kirov A. Crystallography online: Bilbao crystallographic server. Bulg. Chem. Commun. 2011, 43, 183–197.

[ref36] Aroyo M. I.; Perez-Mato J. M.; Capillas C.; Kroumova E.; Ivantchev S.; Madariaga G.; Kirov A.; Wondratschek H. Bilbao Crystallographic Server: I. Databases and crystallographic computing programs. Z. Kristallogr. - Cryst. Mater. 2006, 221, 15–27. 10.1524/zkri.2006.221.1.15.

[ref37] Aroyo M. I.; Kirov A.; Capillas C.; Perez-Mato J. M.; Wondratschek H. Bilbao Crystallographic Server. II. Representations of crystallographic point groups and space groups. Acta Crystallogr., Sect. A: Found. Crystallogr. 2006, 62, 115–128. 10.1107/S0108767305040286. 16489249

[ref38] Kroumova E.; Aroyo M. I.; Perez-Mato J. M.; Kirov A.; Capillas C.; Ivantchev S.; Wondratschek H. Bilbao crystallographic server: useful databases and tools for phase-transition studies. Phase Trans. 2003, 76, 155–170. 10.1080/0141159031000076110.

[ref39] Tian Y.; Gray M. J.; Ji H.; Cava R. J.; Burch K. S. Magneto-elastic coupling in a potential ferromagnetic 2D atomic crystal. 2D Mater. 2016, 3, 025035 10.1088/2053-1583/3/2/025035.

[ref40] Lee J.-U.; Lee S.; Ryoo J. H.; Kang S.; Kim T. Y.; Kim P.; Park C.-H.; Park J.-G.; Cheong H. Ising-type magnetic ordering in atomically thin FePS3. Nano Lett. 2016, 16, 7433–7438. 10.1021/acs.nanolett.6b03052. 27960508

[ref41] Kim K.; Lim S. Y.; Kim J.; Lee J.-U.; Lee S.; Kim P.; Park K.; Son S.; Park C.-H.; Park J.-G.; Cheong H. Antiferromagnetic ordering in van der Waals 2D magnetic material MnPS3 probed by Raman spectroscopy. 2D Mater. 2019, 6, 041001 10.1088/2053-1583/ab27d5.

[ref42] McCreary A.; Simpson J. R.; Mai T. T.; McMichael R. D.; Douglas J. E.; Butch N.; Dennis C.; Valdés Aguilar R.; Hight Walker A. R. Quasi-two-dimensional magnon identification in antiferromagnetic FePS_3_ via magneto-Raman spectroscopy. Phys. Rev. B 2020, 101, 064416 10.1103/PhysRevB.101.064416. PMC11015466 38616972

[ref43] Zhu S.; Zheng W. Temperature-dependent phonon shifts in van der Waals crystals. J. Phys. Chem. Lett. 2021, 12, 5261–5270. 10.1021/acs.jpclett.1c00947. 34060315

[ref44] Steurer W.; Apfolter A.; Koch M.; Ernst W. E.; Søndergård E.; Manson J. R.; Holst B. Anomalous phonon behavior: blueshift of the surface boson peak in silica glass with increasing temperature. Phys. Rev. Lett. 2008, 100, 135504 10.1103/PhysRevLett.100.135504. 18517967

[ref45] Kargar F.; Barani Z.; Sesing N. R.; Mai T. T.; Debnath T.; Zhang H.; Liu Y.; Zhu Y.; Ghosh S.; Biacchi A. J.; et al. Elemental excitations in MoI_3_ one-dimensional van der Waals nanowires. Appl. Phys. Lett. 2022, 121, 221901 10.1063/5.0129904.

[ref46] McCreary A.; Mai T. T.; Utermohlen F. G.; Simpson J. R.; Garrity K. F.; Feng X.; Shcherbakov D.; Zhu Y.; Hu J.; Weber D.; et al. Distinct magneto-Raman signatures of spin-flip phase transitions in CrI3. Nat. Commun. 2020, 11, 3879 10.1038/s41467-020-17320-3. 32747673 PMC7398929

