# Supplementary material for: Bridging Structure, Magnetism, and Disorder in Iron-Intercalated Niobium Diselenide, FexNbSe2, below x = 0.25
Source: J Phys Chem C Nanomater Interfaces. 2023 May 10;127(20):9787–95. doi: 10.1021/acs.jpcc.3c00870 (PMC10226111; doi:10.1021/acs.jpcc.3c00870)
Supplement: Supplementary file 1 — jp3c00870_si_001.pdf [file jp3c00870_si_001.pdf]

Supporting information for

Bridging Structure, Magnetism, and Disorder in Iron-Intercalated Niobium Diselenide, Fe_xNbSe_2, below $x = 0.25$

Matthew P. Erodici[1], Thuc T. Mai[2], Lilia S. Xie[1], Simon Li[1], Shannon S. Fender[1], Samra Husremović[1], Oscar Gonzalez[1], Angela R. Hight Walker[2], and D. Kwabena Bediako[1,3]*

[1] *Department of Chemistry, University of California, Berkeley, California 94720, United States*

[2] *National Institute of Standards and Technology, Gaithersburg, Maryland 20899, United States*

[3] *Chemical Sciences Division, Lawrence Berkeley National Laboratory, Berkeley, California 94720, United States*

**Correspondence to: bediako@berkeley.edu*

Contents

Figure S1. Magnetization vs. field data for Fe_xNbSe_2 ($x = 0.14, 0.19, 0.23$) 1

Figure S2. In-plane vs. out-of-plane magnetic susceptibility data for $Fe_{0.23}NbSe_2$ 2

Figure S3. Thermoremanent magnetization (TRM) measurements for $Fe_{0.14}NbSe_2$ 2

Figure S4. Raman spectra for 2*H*-$NbSe_2$ and $Fe_{0.23}NbSe_2$ with 532 nm vs. 633 nm excitation ... 3

Figure S5. Variable-temperature Raman data for $Fe_{0.23}NbSe_2$ (stacked line plots) 3

Supplementary Note S6. Single crystal data for $Fe_{0.14}NbSe_2$ 4

Supplementary Note S7. Single crystal data for $Fe_{0.19}NbSe_2$ 4

Supplementary Note S8. Single crystal data for $Fe_{0.23}NbSe_2$ 4

Table S9. Bond lengths for $Fe_{0.23}NbSe_2$ single crystal 4

Table S10. Bond angles for $Fe_{0.23}NbSe_2$ single crystal 5

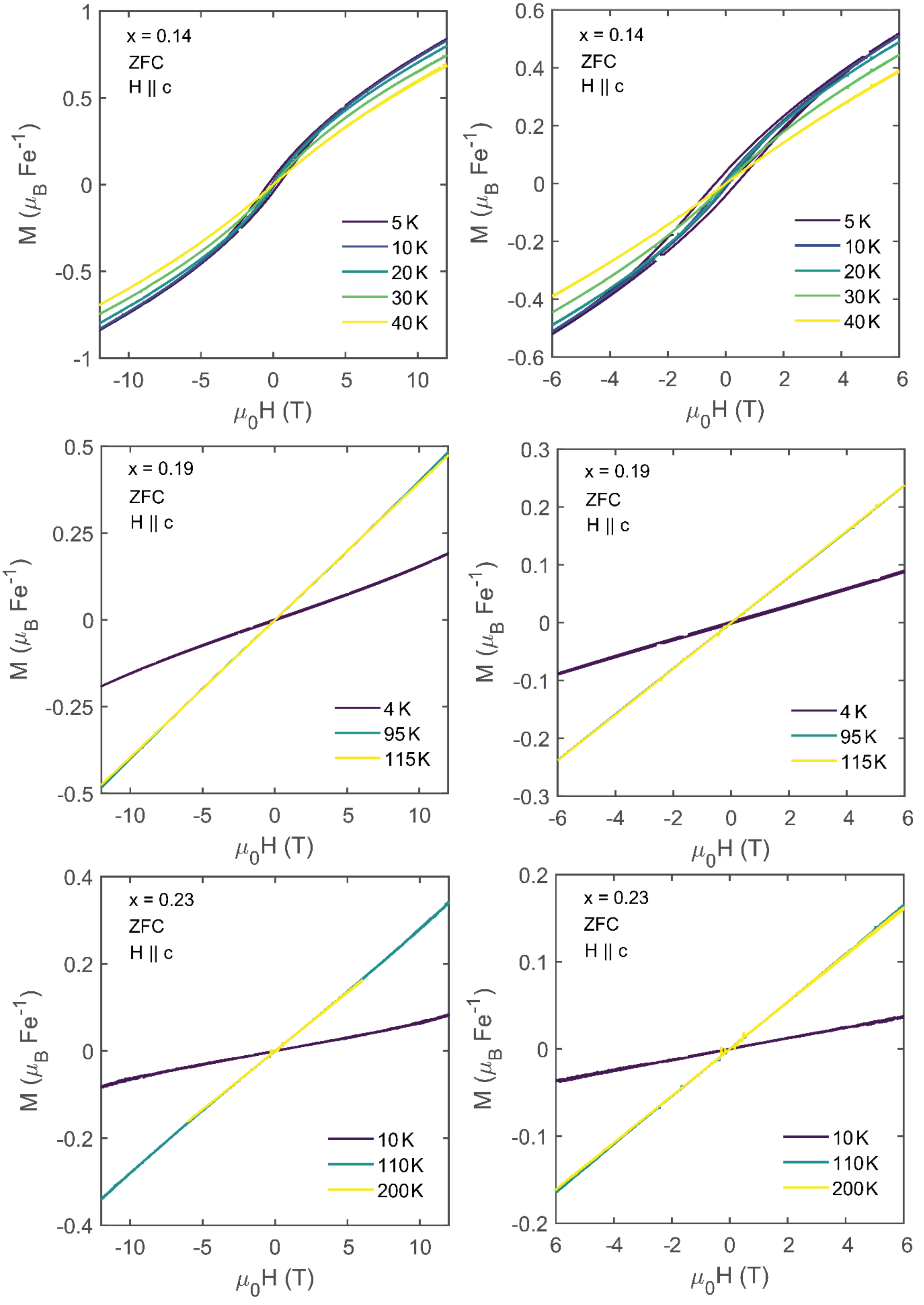

Figure S1. Magnetization vs. field data for Fe_xNbSe_2 crystals (x = 0.14, 0.19, 0.23) with magnetic field applied along the c crystallographic axis.

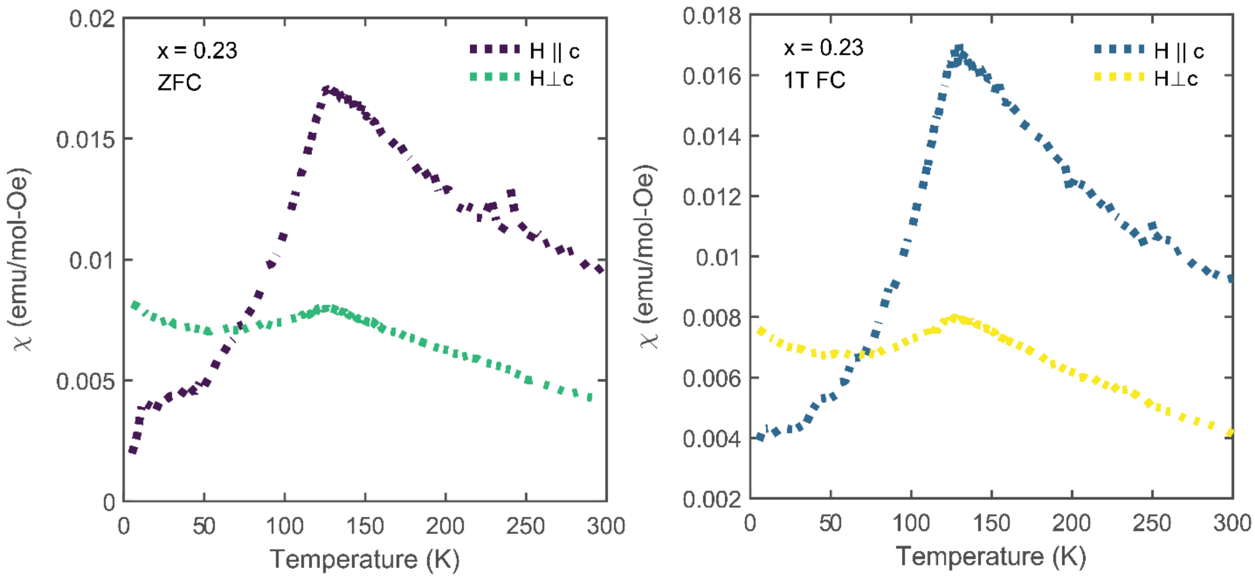

Figure S2. In-place vs. out-of-plane magnetic susceptibility (χ) data upon warming for $Fe_{0.23}NbSe_2$ crystals measured after zero-field cooling (ZFC) and 1T-field cooling (FC). The decrease in susceptibility upon applying an in-plane magnetic field (i.e. perpendicular to the *c* crystallographic axis) indicates strong magnetocrystalline anisotropy with preference for ordering out-of-plane.

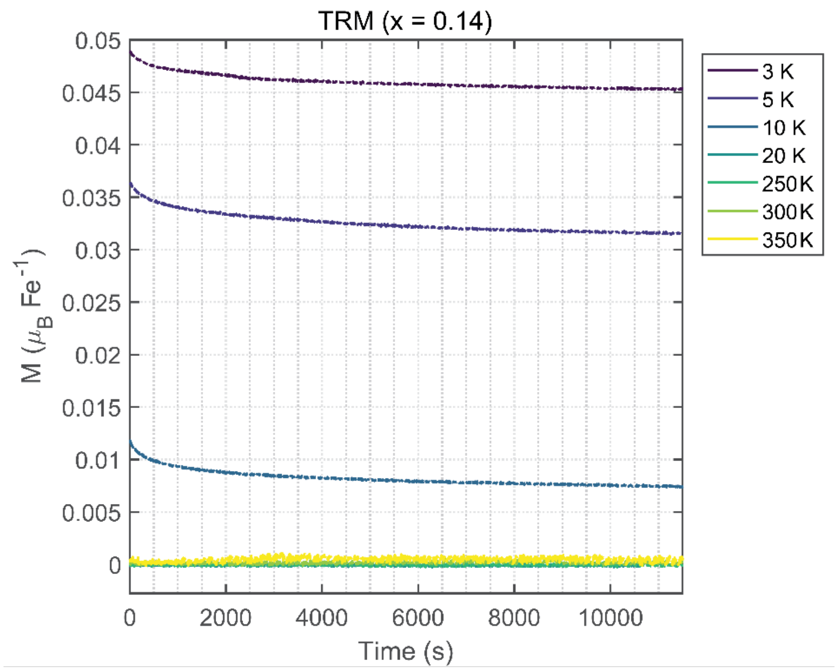

Figure S3. Thermoremanent magnetization (TRM) measurements for the $Fe_{0.14}NbSe_2$ crystal. Before $t = 0$ s, the sample is held in a 1 T magnetic field (applied along the *c* crystallographic axis) for one hour. At $t = 0$ s, the field is ramped down to 0 T, and the remanent magnetization in the crystal is measured. For a sample in the paramagnetic regime, there should be zero remanent magnetization once the field is removed (based on $M = \chi H$), which is the case for measurements at 20 K and above. For a sample in a spin-glass phase, an initial nonzero remanent magnetization is expected—which should decay to a steady-state value over time due to glassy dynamics—which is the case for measurements at 10 K and below.

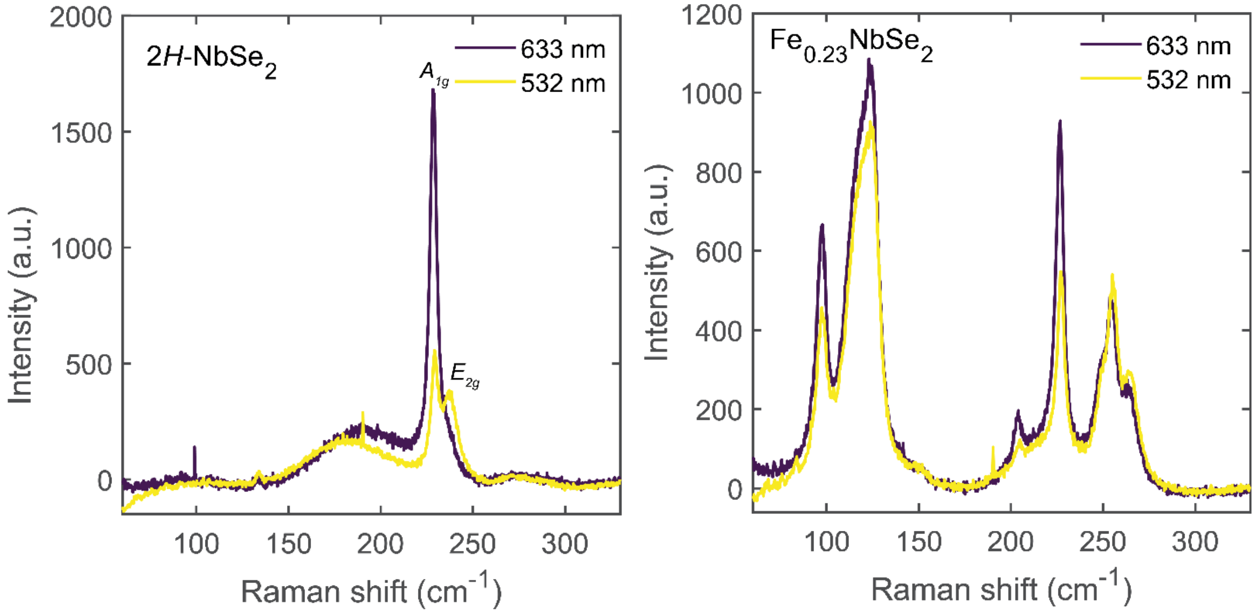

Figure S4. Raman spectra of $NbSe_2$ and $Fe_{0.23}NbSe_2$ crystals taken under ambient conditions and acquired with both 532 and 633 nm excitation. With 532 nm excitation, the expected E_{2g} mode at 237 cm^{-1} can be observed for 2*H*-$NbSe_2$. There are no significant differences or appearance of new modes in $Fe_{0.23}NbSe_2$ when using 532 nm excitation, suggesting no resonance Raman effects present with either excitation wavelength.

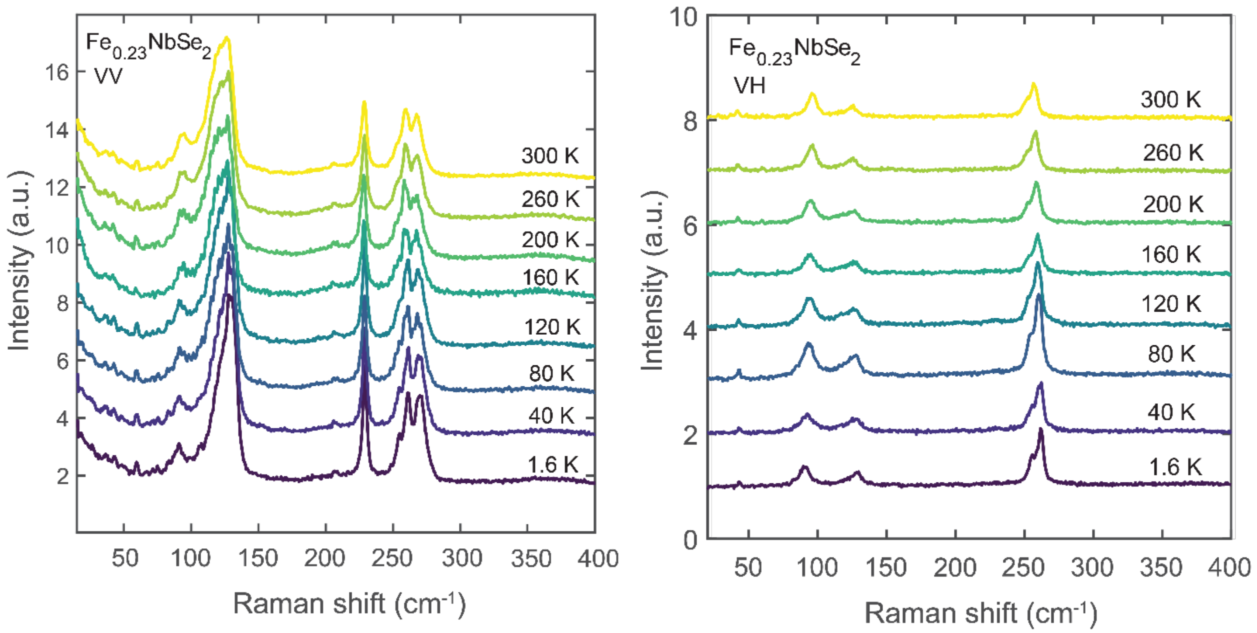

Figure S5. Stacked line plots of representative variable temperature Raman data for $Fe_{0.23}NbSe_2$ single crystal taken in parallel (VV) and cross (VH) polarization configurations.

Supplementary Note S6. Crystal Data for $Fe_{0.14}NbSe_2$: hexagonal, space group P6$_3$/mmc (no. 194), $a = b$ = 3.4490(0) Å, c = 12.6011(9) Å, V = 129.815(1) Å^3, Z = 2, T = 293 K, μ(Mo Kα) = 115.657 mm^{-1}, *Dcalc* = 23.174 g/cm^3, 2752 reflections measured (6.466° ≤ 2Θ ≤ 100.916°), 314 unique (R_{int} = 0.0450, R_{sigma} = 0.0259) which were used in all calculations. The final R_1 was 0.0428 (I > 2σ(I)) and wR_2 was 0.1141 (all data).

Supplementary Note S7. Crystal Data for $Fe_{0.19}NbSe_2$: hexagonal, space group P6$_3$/mmc (no. 194), $a = b$ = 6.9046(3) Å, c = 12.6174(8) Å, V = 520.93(6) Å^3, Z = 2, T = 293 K, μ(Mo Kα) = 20.778 mm^{-1}, *Dcalc* = 4.176 g/cm^3, 777 reflections measured (6.458° ≤ 2Θ ≤ 86.048°), 777 unique (R_{int} = ?, R_{sigma} = 0.0306) which were used in all calculations. The final R_1 was 0.0859 (I > 2σ(I)) and wR_2 was 0.2812 (all data).

Supplementary Note S8. Crystal Data for $Fe_{0.23}NbSe_2$: hexagonal, space group P6$_3$/mmc (no. 194), $a = b$ = 6.9151(4) Å, c = 12.6498(8) Å, V = 523.86(7) Å^3, Z = 2, T = 293 K, μ(Mo Kα) = 33.326 mm^{-1}, *Dcalc* = 6.715 g/cm^3, 5690 reflections measured (6.442° ≤ 2Θ ≤ 59.59°), 314 unique (R_{int} = 0.1208, R_{sigma} = 0.0388) which were used in all calculations. The final R_1 was 0.0641 (I > 2σ(I)) and wR_2 was 0.1484 (all data).

Table S9 Bond Lengths for $Fe_{0.23}NbSe_2$ single crystal.

Atom	Atom	Length/Å	Atom	Atom	Length/Å
Nb01	Se02^1	2.6112(8)	Nb03	Se02^9	2.6144(7)
Nb01	Se02^2	2.6112(8)	Nb03	Nb03^{10}	3.3177(15)
Nb01	Se02^3	2.6112(8)	Nb03	Nb03^{11}	3.3177(16)
Nb01	Se02^4	2.6112(8)	Nb03	Se1	2.5789(11)
Nb01	Se02^5	2.6112(8)	Nb03	Se1^5	2.5789(11)
Nb01	Se02	2.6112(8)	Se1	Nb03^{11}	2.5789(11)
Nb01	Fe05^6	3.1624(2)	Se1	Nb03^{10}	2.5789(11)
Nb01	Fe05	3.1624(2)	Fe05	Nb01^{12}	3.1624(2)
Se02	Nb03^7	2.6144(7)	Fe05	Se02^4	2.5234(8)
Se02	Nb03	2.6144(7)	Fe05	Se02^{13}	2.5234(8)
Se02	Fe05	2.5234(8)	Fe05	Se02^{14}	2.5234(8)
Nb03	Se02^8	2.6144(7)	Fe05	Se02^2	2.5234(8)
Nb03	Se02^5	2.6144(7)	Fe05	Se02^{12}	2.5234(8)

^{1}(2-Y,1+X-Y,1/2-Z); ^{2}(1+Y-X,2-X,+Z); ^{3}(1+Y-X,2-X,1/2-Z); ^{4}(2-Y,1+X-Y,+Z); ^{5}(+X,+Y,1/2-Z); ^{6}(2-X,2-Y,-1/2+Z); ^{7}(2-Y,2+X-Y,+Z); ^{8}(+Y-X,2-X,+Z); ^{9}(+Y-X,2-X,1/2-Z); ^{10}(+Y-X,1-X,+Z); ^{11}(1-Y,1+X-Y,+Z); ^{12}(2-X,2-Y,1-Z); ^{13}(1-Y+X,+X,1-Z); ^{14} (+Y,1-X+Y,1-Z)

Table S10 Bond Angles for $Fe_{0.23}NbSe_2$ single crystal.

Atom	Atom	Atom	Angle/°	Atom	Atom	Atom	Angle/°
Se02^1	Nb01	Se02^2	134.447(14)	Se02^9	Nb03	Nb03^{11}	92.02(2)
Se02^2	Nb01	Se02^3	78.52(4)	Nb03^{11}	Nb03	Nb03^{10}	60.0
Se02^2	Nb01	Se02^4	84.22(3)	Se1	Nb03	Se02^8	83.51(3)
Se02^3	Nb01	Se02^4	134.447(14)	Se1	Nb03	Se02^5	83.51(3)

Table S10 Bond Angles for Fe$_{0.23}$NbSe$_2$ single crystal.

Atom	Atom	Atom	Angle/°	Atom	Atom	Atom	Angle/°
Se02^{5}	Nb01	Se02^{3}	84.22(3)	Se1^{5}	Nb03	Se02	83.51(3)
Se02^{1}	Nb01	Se02^{4}	78.52(4)	Se1^{5}	Nb03	Se02^{5}	137.17(2)
Se02^{1}	Nb01	Se02^{5}	84.22(3)	Se1	Nb03	Se02^{9}	137.17(2)
Se02^{5}	Nb01	Se02^{4}	134.447(14)	Se1^{5}	Nb03	Se02^{9}	83.51(3)
Se02^{1}	Nb01	Se02	134.446(14)	Se1	Nb03	Se02	137.17(2)
Se02^{2}	Nb01	Se02	84.22(3)	Se1^{5}	Nb03	Se02^{8}	137.17(2)
Se02^{2}	Nb01	Se02^{5}	134.447(14)	Se1	Nb03	Nb03^{11}	49.966(19)
Se02^{3}	Nb01	Se02	134.447(14)	Se1	Nb03	Nb03^{10}	49.966(19)
Se02^{4}	Nb01	Se02	84.22(3)	Se1^{5}	Nb03	Nb03^{11}	49.966(19)
Se02^{1}	Nb01	Se02^{3}	84.22(3)	Se1^{5}	Nb03	Nb03^{10}	49.967(19)
Se02^{5}	Nb01	Se02	78.52(4)	Se1	Nb03	Se1^{5}	84.07(5)
Se02^{1}	Nb01	Fe05^{6}	50.74(2)	Nb03	Se1	Nb03^{11}	80.07(4)
Se02	Nb01	Fe05	50.74(2)	Nb03	Se1	Nb03^{10}	80.07(4)
Se02^{3}	Nb01	Fe05^{6}	50.74(2)	Nb03^{11}	Se1	Nb03^{10}	80.07(4)
Se02	Nb01	Fe05^{6}	129.26(2)	Nb01^{12}	Fe05	Nb01	180.0
Se02^{4}	Nb01	Fe05	50.74(2)	Se02^{12}	Fe05	Nb01^{12}	53.25(2)
Se02^{5}	Nb01	Fe05	129.26(2)	Se02^{13}	Fe05	Nb01	126.75(2)
Se02^{2}	Nb01	Fe05^{6}	129.26(2)	Se02	Fe05	Nb01	53.25(2)
Se02^{3}	Nb01	Fe05	129.26(2)	Se02^{2}	Fe05	Nb01^{12}	126.75(2)
Se02^{5}	Nb01	Fe05^{6}	50.74(2)	Se02^{4}	Fe05	Nb01^{12}	126.75(2)
Se02^{4}	Nb01	Fe05^{6}	129.26(2)	Se02^{14}	Fe05	Nb01	126.75(2)
Se02^{2}	Nb01	Fe05	50.74(2)	Se02^{14}	Fe05	Nb01^{12}	53.25(2)
Se02^{1}	Nb01	Fe05	129.26(2)	Se02^{2}	Fe05	Nb01	53.25(2)
Fe05^{6}	Nb01	Fe05	180.0	Se02^{13}	Fe05	Nb01^{12}	53.25(2)
Nb01	Se02	Nb03^{7}	82.88(3)	Se02^{4}	Fe05	Nb01	53.25(2)
Nb01	Se02	Nb03	82.88(3)	Se02^{12}	Fe05	Nb01	126.75(2)
Nb03^{7}	Se02	Nb03	86.94(5)	Se02	Fe05	Nb01^{12}	126.75(2)
Fe05	Se02	Nb01	76.014(18)	Se02^{12}	Fe05	Se02^{2}	92.12(3)
Fe05	Se02	Nb03	131.60(2)	Se02^{4}	Fe05	Se02^{12}	92.12(3)
Fe05	Se02	Nb03^{7}	131.60(2)	Se02^{13}	Fe05	Se02^{4}	180.0
Se02^{8}	Nb03	Se02	130.64(5)	Se02^{12}	Fe05	Se02^{14}	87.88(3)
Se02^{8}	Nb03	Se02^{5}	81.50(4)	Se02	Fe05	Se02^{12}	180.0
Se02	Nb03	Se02^{5}	78.41(4)	Se02^{13}	Fe05	Se02^{14}	87.88(3)
Se02^{9}	Nb03	Se02^{5}	130.64(5)	Se02^{13}	Fe05	Se02^{12}	87.88(3)
Se02^{9}	Nb03	Se02	81.50(4)	Se02	Fe05	Se02^{14}	92.12(3)
Se02^{9}	Nb03	Se02^{8}	78.41(4)	Se02^{13}	Fe05	Se02^{2}	92.12(3)
Se02^{5}	Nb03	Nb03^{10}	92.02(2)	Se02^{4}	Fe05	Se02^{2}	87.88(3)
Se02^{8}	Nb03	Nb03^{11}	92.02(2)	Se02^{4}	Fe05	Se02	87.87(3)
Se02	Nb03	Nb03^{10}	92.02(2)	Se02	Fe05	Se02^{2}	87.88(3)
Se02^{9}	Nb03	Nb03^{10}	133.47(2)	Se02^{4}	Fe05	Se02^{14}	92.12(3)
Se02^{8}	Nb03	Nb03^{10}	133.47(2)	Se02^{13}	Fe05	Se02	92.13(3)
Se02	Nb03	Nb03^{11}	133.47(2)	Se02^{14}	Fe05	Se02^{2}	180.0
Se02^{5}	Nb03	Nb03^{11}	133.47(2)				

[1](2-Y,1+X-Y,1/2-Z); [2](1+Y-X,2-X,+Z); [3](1+Y-X,2-X,1/2-Z); [4](2-Y,1+X-Y,+Z); [5](+X,+Y,1/2-Z); [6](2-X,2-Y,-1/2+Z); [7](2-Y,2+X-Y,+Z); [8](+Y-X,2-X,+Z); [9](+Y-X,2-X,1/2-Z); [10](+Y-X,1-X,+Z); [11](1-Y,1+X-Y,+Z); [12](2-X,2-Y,1-Z); [13](1-Y+X,+X,1-Z); [14] (+Y,1-X+Y,1-Z)
